# Dendritic cells provide a therapeutic target for synthetic small molecule analogues of the parasitic worm product, ES-62

**DOI:** 10.1038/s41598-017-01651-1

**Published:** 2017-05-10

**Authors:** Felicity E. Lumb, James Doonan, Kara S. Bell, Miguel A. Pineda, Marlene Corbet, Colin J. Suckling, Margaret M. Harnett, William Harnett

**Affiliations:** 1 Strathclyde Institute of Pharmacy and Biomedical Sciences, University of Strathclyde, Glasgow, UK; 2 Institute of Infection, Immunity and Inflammation, University of Glasgow, Glasgow, UK; 3 Department of Pure & Applied Chemistry, University of Strathclyde, Glasgow, UK

## Abstract

ES-62, a glycoprotein secreted by the parasitic filarial nematode *Acanthocheilonema viteae*, subverts host immune responses towards anti-inflammatory phenotypes by virtue of covalently attached phosphorylcholine (PC). The PC dictates that ES-62 exhibits protection in murine models of inflammatory disease and hence a library of drug-like PC-based small molecule analogues (SMAs) was synthesised. Four sulfone-containing SMAs termed 11a, 11e, 11i and 12b were found to reduce mouse bone marrow-derived dendritic cell (DC) pathogen-associated molecular pattern (PAMP)-induced pro-inflammatory cytokine production, inhibit NF-κB p65 activation, and suppress LPS-induced up-regulation of CD40 and CD86. Active SMAs also resulted in a DC phenotype that exhibited reduced capacity to prime antigen (Ag)-specific IFN-γ production during co-culture with naïve transgenic TCR DO.11.10 T cells *in vitro* and reduced their ability, following adoptive transfer, to prime the expansion of Ag-specific T lymphocytes, specifically T_H_17 cells, *in vivo*. Consistent with this, mice receiving DCs treated with SMAs exhibited significantly reduced severity of collagen-induced arthritis and this was accompanied by a significant reduction in IL-17^+^ cells in the draining lymph nodes. Collectively, these studies indicate that drug-like compounds that target DCs can be designed from parasitic worm products and demonstrate the potential for ES-62 SMA-based DC therapy in inflammatory disease.

## Introduction

Dendritic cells (DCs) are professional antigen (Ag)-presenting cells that provide a critical link between the innate and adaptive immune systems. They recognise pathogen-associated molecular patterns (PAMPs), as well as host danger signals, through conserved pattern recognition receptors (PRRs) such as the Toll-like receptors (TLRs)^1^. Upon recognition of Ag, DCs leave the peripheral tissue and migrate to the lymph nodes (LNs) where they initiate an adaptive immune response via their interactions with naïve T cells. The type of T cell response generated depends on the secondary signals provided by the DC. Thus, TLR recognition of bacterial or viral ligands induces DC maturation which results in the priming of T helper cell 1 (T_H_1)/T_H_17 responses through secretion of pro-inflammatory cytokines such as IL-12, IL-6 and TNF-α, and up-regulation of co-stimulatory molecules including MHC II, CD40, CD80 and CD86^2^. Recognition of parasitic helminth molecules by DCs does not induce such classical activation, but rather promotes a phenotype that more closely resembles that of immature DCs, characterised by low expression of co-stimulatory molecules and muted secretion of pro-inflammatory cytokines. Moreover, these DCs are refractory to subsequent classical activation through stimulation with TLR ligands such as LPS^3^. Nevertheless, such DCs still appear capable of generating strong T_H_2 responses, which may be accompanied by induction of a regulatory T cell (Treg) phenotype^4, 5^.

ES-62, a glycoprotein secreted by the rodent filarial nematode *Acanthocheilonema viteae*, was the first parasitic helminth molecule found to modulate the ability of DCs to prime polarised T_H_ responses when it was shown that DCs matured with ES-62 promote Ag-specific IL-4 production by T cells whilst inhibiting their capacity to produce IFN-γ *in vitro* ^6^. This reflects that DCs exposed to ES-62 *in vitro* or *in vivo*, exhibit decreased capacity to generate pro-inflammatory responses to TLR2, 4 and 9 ligands (BLP, LPS and CpG), as evidenced by their reduced production of pro-inflammatory cytokines such as IL-12 and TNF-α^7^. Consistent with this ability to suppress such pro-inflammatory responses, ES-62 has also been demonstrated to be protective in several murine models of T_H_1/T_H_17-mediated inflammatory disease, for example, collagen-induced arthritis (CIA), although in this model ES-62 does not appear to induce compensatory T_H_2 responses^8^. The protective effects raise the possibility of utilising ES-62 therapeutically, but as a large, potentially immunogenic molecule, it is unsuitable for development as a treatment for human disease. However, as post-translational attachment of phosphorylcholine (PC) to *N*-glycan moieties has been shown to be responsible for the majority of ES-62’s anti-inflammatory effects (reviewed in ref. 8), a library of small molecule analogues (SMAs) of ES-62 based around PC has been designed and synthesised with the aim of finding the best way of presenting this immunomodulatory component in drug-format.

Two of these SMAs, sulfone compounds termed 11a and 12b, have previously been demonstrated to modulate macrophage and mast cell responses and, as with ES-62, to protect against development of CIA in mice^9–11^. Given the pivotal role of DCs in the protection afforded by ES-62 in suppressing IL-17 responses in CIA^12^, we wished to determine whether DCs were a therapeutic target for the SMAs: we now show that SMAs 11a and 12b, along with two other sulfones - 11e and 11i, indeed modulate DC responses *in vitro* such that the cells are refractory to subsequent activation by TLR-PAMPs and are similarly characterised by their reduced expression of co-stimulatory molecules CD40 and CD86, inhibited production of IL-12, IL-6 and TNF-α, and abrogated priming of Ag-specific T_H_1 responses. The sulfones 11e and 11i, unlike 11a and 12b, have not yet been studied much further than the primary response of the cytokine release profile; 11e and 11i are close structural analogues of 11a having 3- and 4- fluoro substituents in the benzene ring in place of the 4-bromo substituent in 11a. Expanding the structural diversity available in active SMAs is valuable not only from the point of view of scientific understanding but also from the point of view of potential therapeutic applications of SMAs. In addition, we demonstrate that transfer of SMA-treated DCs into mice suppresses Ag-specific clonal expansion and T_H_17 polarisation. Finally, consistent with recent reports that helminth-matured DCs can influence autoimmune disease progression in mouse models^13–15^, simply adoptively transferring DCs treated with 11a plus 12b was found to suppress the severity of development of CIA. As such DCs mimic the previously reported effects in CIA observed when these SMAs were administered directly, these findings support the therapeutic potential of SMA-DC therapy in inflammatory diseases such as RA.

## Results

### Small molecule analogues (SMAs) of ES-62 modulate dendritic cell responses

**Figure 1:**
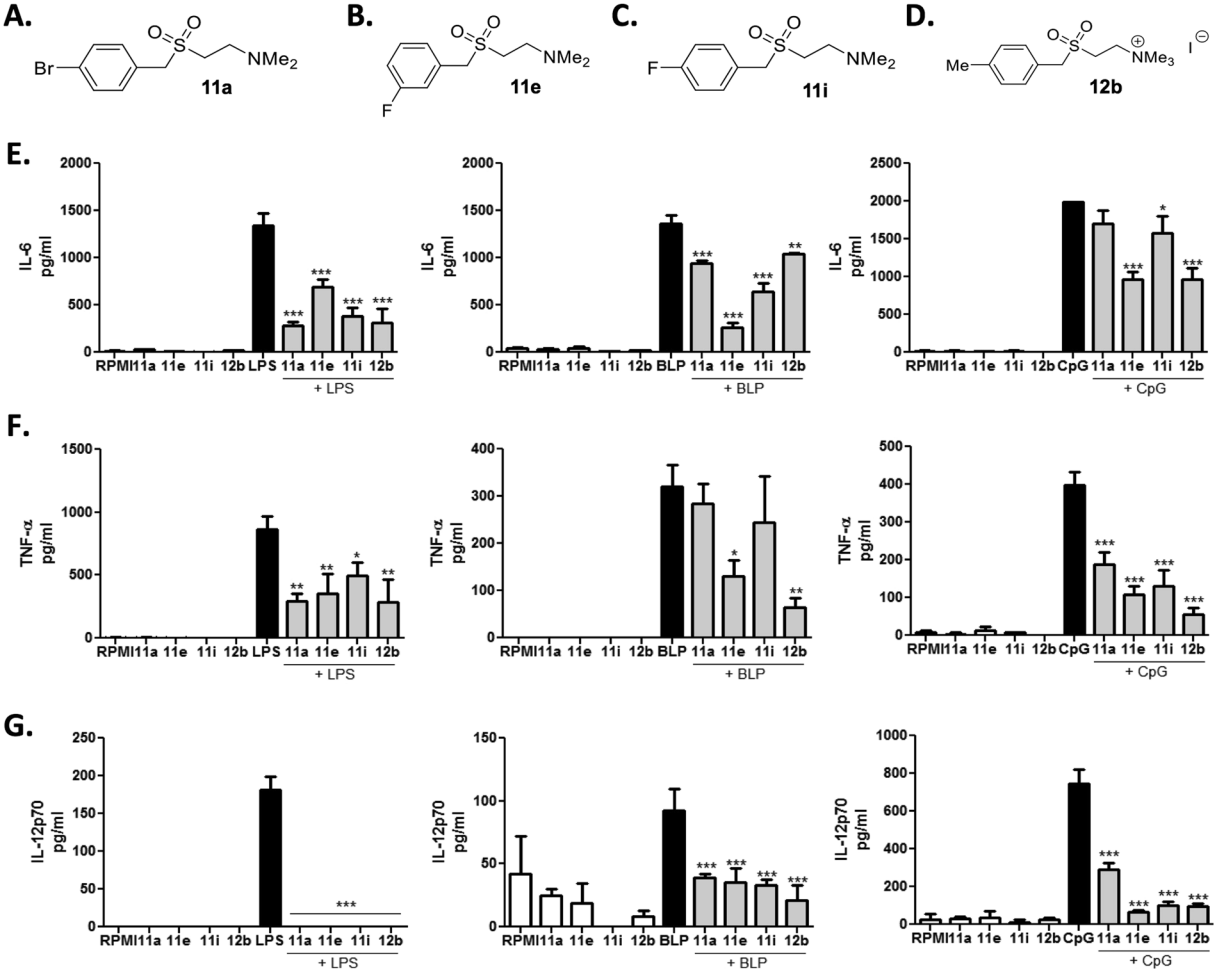
Small molecule analogues (SMAs) of the parasitic worm product ES-62 inhibit PAMP-induced cytokine production. The structures of SMAs 11a (**A**), 11e (**B**), 11i (**C**) and 12b (**D**) are shown. BmDCs were incubated with SMA (5 μg/ml) for 18 hours before stimulation with LPS, BLP (both 100 ng/ml) or CpG (0.1 μM) and the production of IL-6 (**E**) TNF-α (**F**) and IL-12p70 (**G**) was measured by ELISA. Data are expressed as means (of triplicate determinations) ± SD and analysed using one-way ANOVA with Bonferroni post-test where *p < 0.05; **p < 0.01; ***p < 0.001 compared to relevant PAMP. All data are representative of at least two independent experiments.

The library (79 SMAs^9^) was initially screened *in vitro* to determine the effect of SMAs on subsequent PAMP-mediated pro-inflammatory cytokine production by bmDCs (Supplementary Table 1) in order to identify any SMAs that could mimic the previously reported modulatory effects of ES-62 (reviewed by ref. 16). Sulfone-type SMAs 11a, 11e, 11i and 12b (structures shown in Fig. 1A–D) each reduced the LPS-stimulated production of IL-6, TNF-α (although this did not reach statistical significance in all experiments with 11a) and IL-12(p70) (Fig. 1E–G, left hand panels). It was next investigated whether these SMAs were also capable of inhibiting the cytokine responses induced by ligands of TLR2 and TLR9 as both of these PRRs, along with TLR3 and TLR4, are thought to play an important role in the pathogenesis of RA^17, 18^. SMAs 11e and 12b significantly inhibited BLP- and CpG-induced IL-6, TNF-α and IL-12(p70) secretion while 11i significantly inhibited all of these responses except BLP-induced TNF-α (Fig. 1E–G, middle and right hand panels). SMA 11a demonstrated the most selectivity as it did not inhibit BLP-induced TNF-α and CpG-induced IL-6 but all other responses were reduced (Fig. 1E–G, middle and right hand panels). However, none of the SMAs inhibited the production of IL-12p40; indeed, 11a significantly increased the BLP- and LPS-induced production of this cytokine by bmDCs (data not shown), suggesting that as with ES-62, IL-12p35 may be the major target^19^ in the suppression of IL-12p70. Overall, the observed selectivity in response was not found when testing the SMAs on macrophages^9^ suggesting that the compounds may exhibit differential effects in distinct cell types.

**Figure 2:**
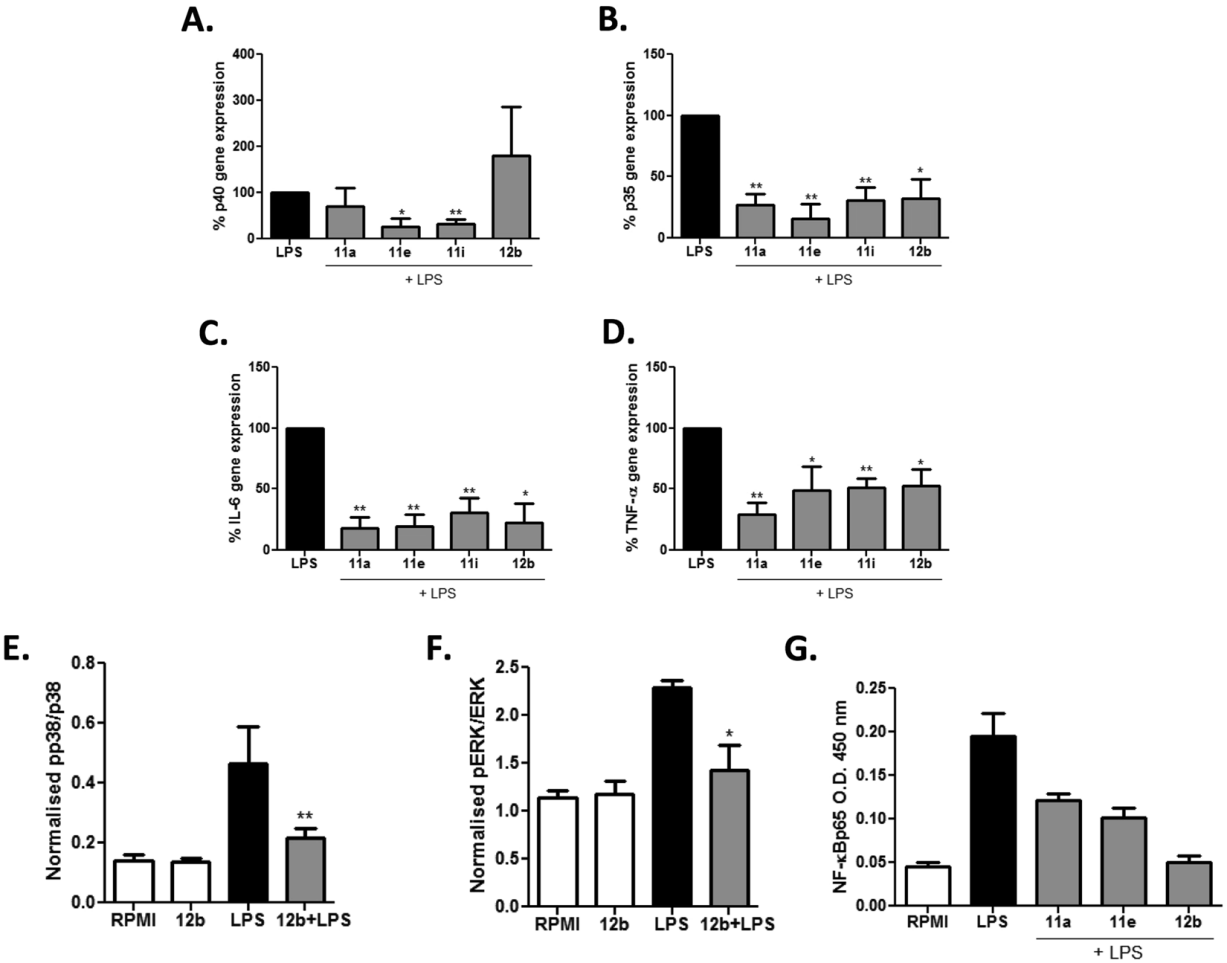
ES-62 SMAs target cytokine production at the level of transcription and display differing effects on the activation of MAPKs and NF-κBp65. BmDCs were treated with SMAs 11a, 11e, 11i or 12b (5 μg/ml) for 18 hours, matured with LPS for 4 h, before extracting RNA and analysing the expression levels of IL-12p40 (**A**), IL-12p35 (**B**), IL-6 (**C**) and TNF-α (**D**) by qRT-PCR. Cytokine expression was normalised to GAPDH and the levels of mRNA in SMA + LPS (from all experiments [n = 3]) compared to LPS (100%) DC calculated and compared using a one sample t test. Results are expressed as the mean ± SEM. BmDCs were treated with SMA 12b for 18 hours and then stimulated with LPS for 10 minutes and the levels of total and phosphorylated p38 (**E**) and ERK (**F**) measured by fast-activated cell ELISA (FACE). Phosphorylated p38 or ERK1/2 absorbance were normalised to the corresponding total p38 or ERK1/2 absorbance and the data expressed as means (of triplicate determinations) ± SD. Data were analysed using one way ANOVA with Bonferroni post-test where for statistical analysis, *p < 0.05; **p < 0.01. As above, bmDCs were incubated with SMAs for 18 hours before stimulation with LPS for 30 minutes and the level of p65 activation in duplicate samples measured by TransAM (**G**). All data are representative of at least two independent experiments.

As the SMAs inhibited IL-12p70 but not IL-12p40 following PAMP stimulation we next investigated whether IL-12p35 and IL-12p40, were differentially regulated at the mRNA level by the SMAs. Consistent with the protein secretion data, whilst SMAs 11a and 12b had no significant inhibitory effect on the LPS-induced levels of p40, and in fact increased the basal levels (data not shown), they significantly reduced LPS-induced p35 expression (Fig. 2A,B). However, SMAs 11e and 11i significantly inhibited the LPS-stimulated expression of both IL-12 subunits (Fig. 2A,B) as well as the basal levels of IL-12p35 but not IL-12p40 (data not shown). In addition, all four SMAs significantly inhibited the level of LPS-induced IL-6 and TNF-α gene expression indicating they likely target these cytokines at the transcription/message stability level (Fig. 2C,D).

ES-62 differentially targets ERK and p38 MAPK signalling to mediate suppression of IL-12p40 and p35 subunits, with inhibition of p38 resulting in reduced production of p35 (and IL-6) whilst augmentation of ERK-mediated negative feedback mediates suppression of p40 production in LPS + IFNγ–stimulated macrophages^19^. Incubation of bmDCs with SMAs alone did not alter the activation of either p38 or ERK MAPK (data not shown) whereas stimulation of the cells with LPS for 10 min resulted in significant activation of both ERK and p38 MAPK. Consistent with its ability to inhibit IL-12p35 and IL-6 production, pre-treatment with SMA 12b significantly suppressed LPS-stimulated p38 activation but interestingly none of the other SMAs had any significant effects on this MAPK (Fig. 2E and data not shown). Likewise, SMA 12b, but not the other SMAs, caused a reduction in the LPS-mediated ERK activation (Fig. 2F and data not shown).

The suppression of PAMP-induced cytokine release by ES-62 or the SMAs 11a and 12b in macrophages and mast cells is also associated with inhibition of RelA p65 NF-κB^9, 11^, although such an effect of the SMAs in DCs has not previously been investigated. Analysis of the levels of p65 activation in bmDCs after SMA pre-treatment and LPS stimulation in the present study revealed that 11a, 12b and 11e inhibited NF-κB activation as shown when compared to results obtained in response to treatment with LPS alone (Fig. 2G), although SMA 11i had an inconsistent effect on the activation of p65 (data not shown). Treatment with SMAs alone had no effect on the basal activation of p65 NF-κB (data not shown).

### ES-62 SMAs modulate the ability of DCs to drive T_H_1/T_H_17 responses

**Figure 3:**
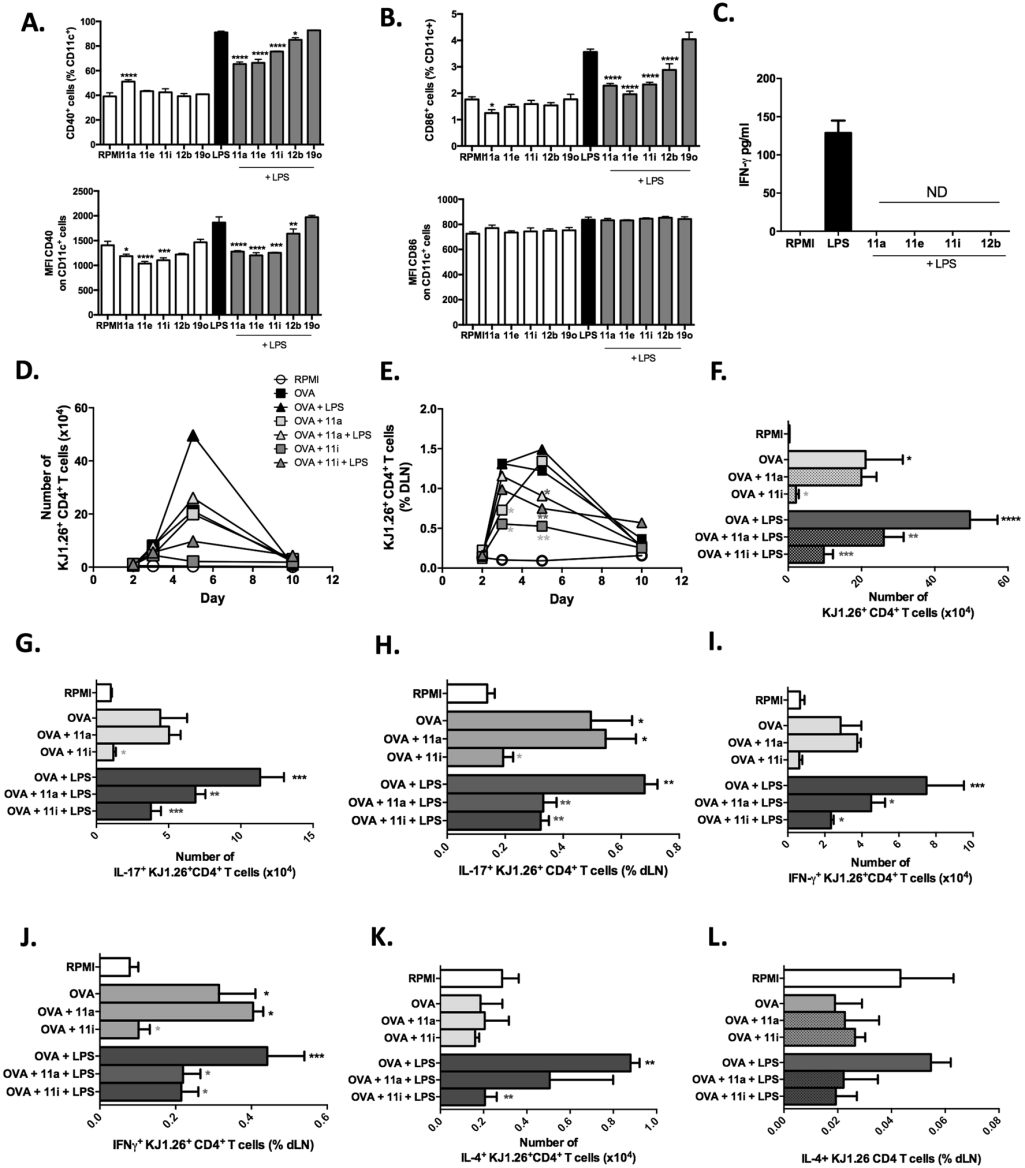
ES-62 SMAs modulate bmDC priming of T cell responses. BmDCs were incubated with SMAs for 18 hours, stimulated with LPS (100 ng/ml) for 24 hours, and CD40 (**A**) and CD86 (**B**) expression by CD11c^+^ cells measured by flow cytometry in term of % cells (upper panels) and Median Fluorescence Intensity (MFI; lower panels). (**C**) BmDCs from BALB/c mice pre-incubated with/without SMAs (5 μg/mL), stimulated for 24 hours with LPS and then pulsed with OVA peptide, were co-cultured with naive OVA-specific CD4^+^ T cells for 3 days before measuring IFN-γ release by ELISA. Inhibitory effects were observed at several peptide concentrations over two independent experiments and the results shown are for 300 μM. Data are presented as means ± SD and were analysed by one way ANOVA with Bonferroni post-test where *p < 0.05; **p < 0.01; ***p < 0.001. For panel (**C**), “ND” = not detected. BmDCs were incubated with/without SMA 11a or 11i and pulsed with OVA overnight before stimulation ± LPS for 24 hours and injected into BALB/c mice that had received 10^6^ naïve CD4^+^KJ1.26^+^ T cells from donor DO11.10 mice 24 h previously. The number (**D**) and percentage (**E**) of KJ1.26^+^ CD4 T cells in the popliteal dLNs of adoptively transferred mice on different days is shown. Data are expressed as the mean percentage of 3 mice per group per day and the error bars (SEM) are omitted for clarity. On days 3 and 5 all treatments except OVA+SMA on day 3 and OVA+11i on day 5 demonstrated a significant increase in the proportion of KJ1.26^+^CD4^+^ T cells compared to RPMI DCs alone. The numbers of these T cells on day 5 (**F**) and their expression of intracellular IL-17 (**G**,**H**), IFNγ (**I**,**J**) and IL-4 (**K**,**L**) on this day were analysed by flow cytometry. Data are expressed as mean ± SEM for 3 mice per group and analysed using Fishers LSD test where *p < 0.05, **p < 0.01 and ***p < 0.001; ****p < 0.0001 compared to RPMI;  p < 0.05 compared to OVA DCs and  p < 0.05 and  p < 0.01 compared to OVA+LPS DCs.

The cytokines produced by DCs and the co-stimulatory molecules that they express when they present Ag to naïve T cells play a key role in the polarisation and development of the subsequent immune response. As we have shown the SMAs to inhibit the pro-inflammatory cytokine production of DCs, it was next investigated whether they also affect their co-stimulatory molecule expression following LPS maturation: indeed, pre-treatment with all four SMAs significantly reduced the LPS-induced up-regulation in the proportion of cells expressing CD40 or CD86 (Fig. 3A,B). In addition, we checked the levels of expression (MFI) and whereas this was also reduced with respect to CD40, this was not the case for CD86 (Fig. 3A,B).

It was next therefore investigated whether SMA-mediated modulation of DC maturation could alter the ability of the cells to prime T cell responses *in vitro*. Treatment of DCs with ES-62 was previously demonstrated to promote the Ag-specific priming of IL-4 release by T cells and inhibit their production of IFN-γ, polarising the cells away from a T_H_1 response^6^. Likewise, ES-62 has been shown to suppress the DC-priming of T_H_17 cells, thereby blocking responses associated with pathogenesis in CIA^12^. Investigation of the SMAs revealed that they similarly suppressed (in this case, completely) the priming of IFN-γ (Fig. 3C) suggesting that SMA-exposure can also modulate the ability of bmDCs to prime T_H_1 cytokine responses in naïve CD4^+^CD62L^+^ T cells. However, IL-17 and IL-4 production could not be detected in these experiments (data not shown).

The physiological relevance of these findings was investigated by determining whether DCs exposed *in vitro* to LPS, SMAs, or SMAs + LPS, primed differential T cell responses *in vivo*. Specifically, medium alone (RPMI)-, LPS-, SMA- or SMA+LPS-exposed DCs were transferred into recipient BALB/c mice 24 h after they had received DO.11.10 T cells expressing the transgenic KJ1.26^+^ TCR specific for OVA_323–339_. We have previously used this model to demonstrate ES-62-mediated suppression of clonal expansion and re-polarisation of the effector function of heterologous antigen-specific T cells away from both T_H_1 and T_H_2 responses *in vivo* ^20, 21^. Moreover, pretreatment with ES-62 was found to suppress the ability of LPS-matured DCs to initiate Ag-specific responses upon adoptive transfer^22^. As shown previously^22^, transfer of OVA peptide-loaded DCs matured with LPS induced high levels of Ag-specific T cell expansion compared to control RPMI DCs at day 5 post inoculation while DCs loaded with OVA peptide alone induced a modest increase in the number of these cells (Fig. 3D). Pre-exposure of DCs to either SMA 11a or 11i before LPS stimulation significantly reduced both the percentage and number of CD4^+^KJ1.26^+^ T cells in the dLNs when compared to those obtained with control OVA + LPS-DCs on this day (Fig. 3E,F). Additionally, mice receiving DCs treated with SMA 11i had a significantly reduced percentage of CD4^+^KJ1.26^+^ T cells compared to mice inoculated with control OVA-DCs on days 3 and 5, and a reduced number of these cells on day 5 (Fig. 3E,F). DCs treated with SMA 11a also had a reduced percentage of CD4+KJ1.26+ cells on day 3 but not day 5, relative to control OVA-DCs (Fig. 3E).

We also examined whether such SMA-exposed DCs and SMA+LPS-exposed DCs altered the polarisation of CD4^+^ T cells away from the T_H_1/T_H_17 responses observed in this *in vivo* setting. Mice adoptively transferred with 11a+LPS-DCs or 11i+LPS-DCs demonstrated significantly reduced levels–both numbers and percentages - of IL-17^+^ and IFN-γ^+^ T cells compared to those with LPS-DCs (Fig. 3G–J). SMA 11i-DC inoculated mice also had significantly lower numbers of IFN-γ^+^ and IL-17^+^ CD4^+^ T cells compared to OVA-DC inoculated mice, although with IFNγ this did not reach statistical significance (Fig. 3G–J). Interestingly, treatment with either SMA (although not always reaching statistical significance) also tended to reduce the (low) percentages and levels of IL-4 cells (Fig. 3K,L) induced by LPS-DCs, indicating that while the SMAs may be able to attenuate T_H_1/T_H_17 responses *in vivo*, like ES-62^12, 23^ they do not tend to induce the opposing T_H_2 phenotype.

### SMA-treated DCs protect against the development of collagen-induced arthritis

The ES-62-mediated protection against CIA is associated with suppression of T_H_1, T_H_17 and IL-17-producing γδ T cell responses and this reflects modulation of DC function, with bmDCs from ES-62-treated CIA mice exhibiting a reduced capacity to produce IL-6, TNF-α and IL-23^12^. Likewise ES-62-treated DCs display a reduced ability to generate OVA-specific T_H_1 and T_H_17 cells and such modulation of DC function was found to be responsible for the ES-62-mediated suppression of IL-17 responses by γδ T cells^12^. Collectively these data suggest that DCs are a central target in ES-62-mediated protection during development of CIA. Thus, as both 11a and 12b have been found to be protective in prophylactic and therapeutic models of CIA^9, 11^ and 11a-modulated DCs were found to inhibit both T_H_1 and T_H_17 responses that are crucial for the development of CIA in the adoptive transfer model, it was decided to compare the effects of DCs treated with SMAs 11a and 12b in combination (“SMA DCs”) on CIA with those of control DCs (“RPMI DCs”) and disease-control CIA mice exposed only to PBS.

**Figure 4:**
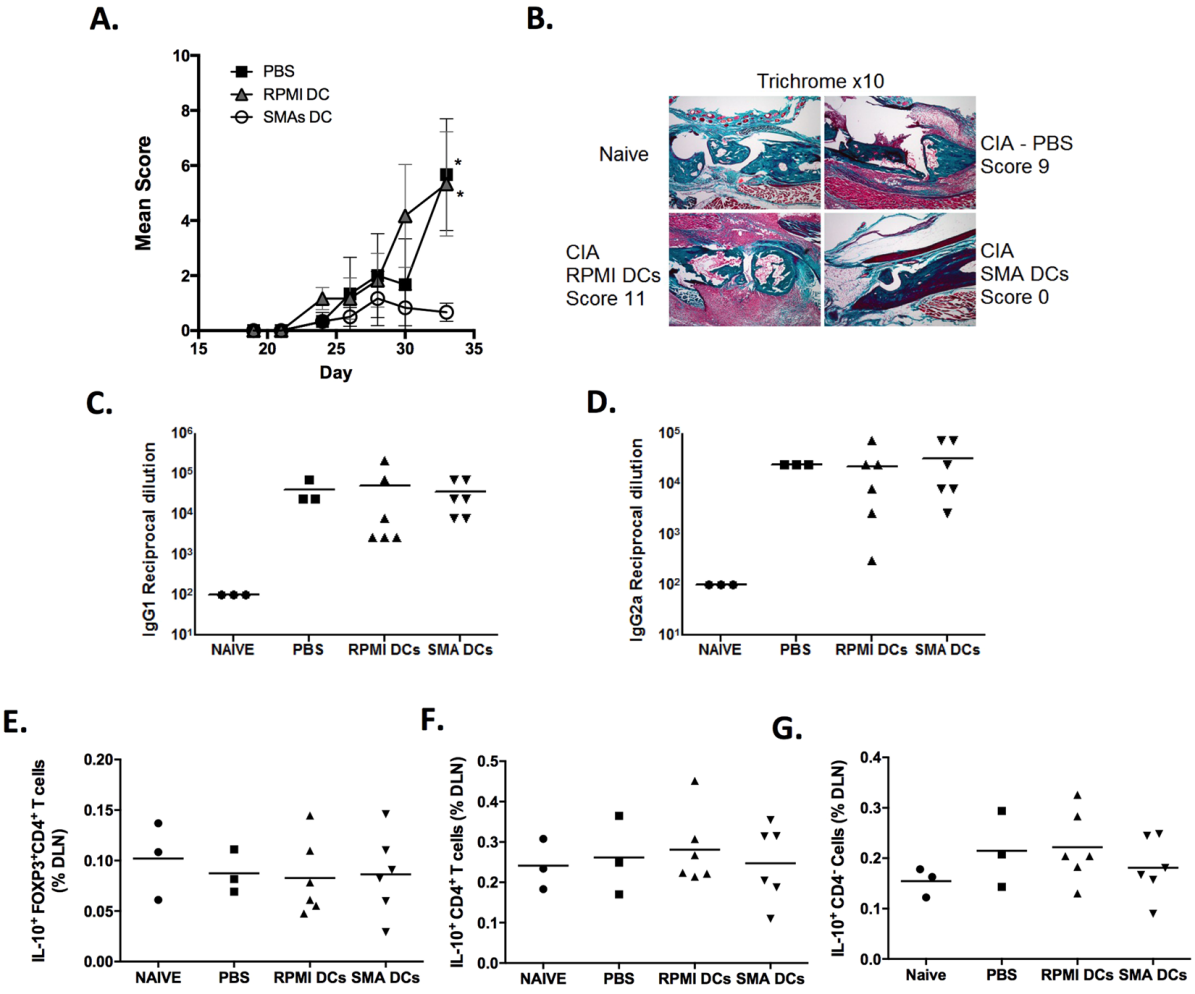
SMA-treated dendritic cells protect against CIA. DBA/1 bmDCs were incubated with SMAs 11a and 12b (2.5 μg/ml of each) and CII overnight, washed, and injected into CIA-mice on day −2, 0 and 21. Disease is shown by mean arthritic score (**A**) (PBS, n = 3; RPMI-DCs and SMA-DCs, n = 6). Data are expressed as means ± SEM and analysed using an unpaired t test where *p < 0.05 compared to PBS. Joint sections (x10 magnification) from individual mice representative of each treatment group were assessed for histopathology by Trichrome staining (**B**). CII-specific IgG1 (**C**) and IgG2a (**D**) levels in serum samples from naïve (no CII treatment; n = 3), PBS- (n = 3), RPMI DC- (n = 6) and SMA DC- (n = 6) treated CIA mice were determined by ELISA. Results are expressed as mean (of duplicate determinations) for each mouse in the group. The percentage of IL-10^+^ FOXP3^+^ CD4^+^ T cells (**E**), IL-10^+^ CD4^+^ T cells (**F**) and CD4^−^IL-10^+^ cells (**G**) in the DLNs from all paws from CIA-mice treated with PBS, RPMI-DCs or SMA-DCs were analysed by flow cytometry. Results from individual mice in each group are shown; numbers as above. Data were analysed using one-way ANOVA employing Fishers LSD test.

Administration of *in vitro* SMA-matured bmDCs but not RPMI DCs, was indeed found to ameliorate CIA as evidenced by reduced arthritic score and suppressed cellular infiltration and damage to the joints (Fig. 4A,B). However, SMA-treated DCs did not modulate the CII-specific antibody responses as the levels of CII-specific IgG1 and IgG2a antibodies were unchanged amongst PBS-, RPMI DC- and SMA DC-inoculated mice (Fig. 4C,D). Previous studies, which have demonstrated protective effects of helminth-matured DCs, have reported that such protection is mediated through the generation of Treg cells and/or the production of IL-10^13–15^. It was therefore investigated whether SMA-matured DCs also mediated protection through these mechanisms. Consistent with studies with ES-62^20, 24^, there was no difference in the percentage of FOXP3^+^CD4^+^ Tregs cells in the dLNs of treated mice relative to the other groups, nor was there any difference in the expression of IL-10 by these cells (Fig. 4E and data not shown). Nor were there any differences amongst the groups in the percentages of CD4^+^IL-10^+^ Tr1 cells (Fig. 4F). While ES-62 does not seem to promote the generation of Tregs it has recently been found that the nematode product restores IL-10-producing B cell levels in CIA-mice back to those present in naïve mice^25^. However, there was also no difference in the levels of IL-10^+^CD4^−^ cells found amongst the treatment groups (Fig. 4G).

**Figure 5:**
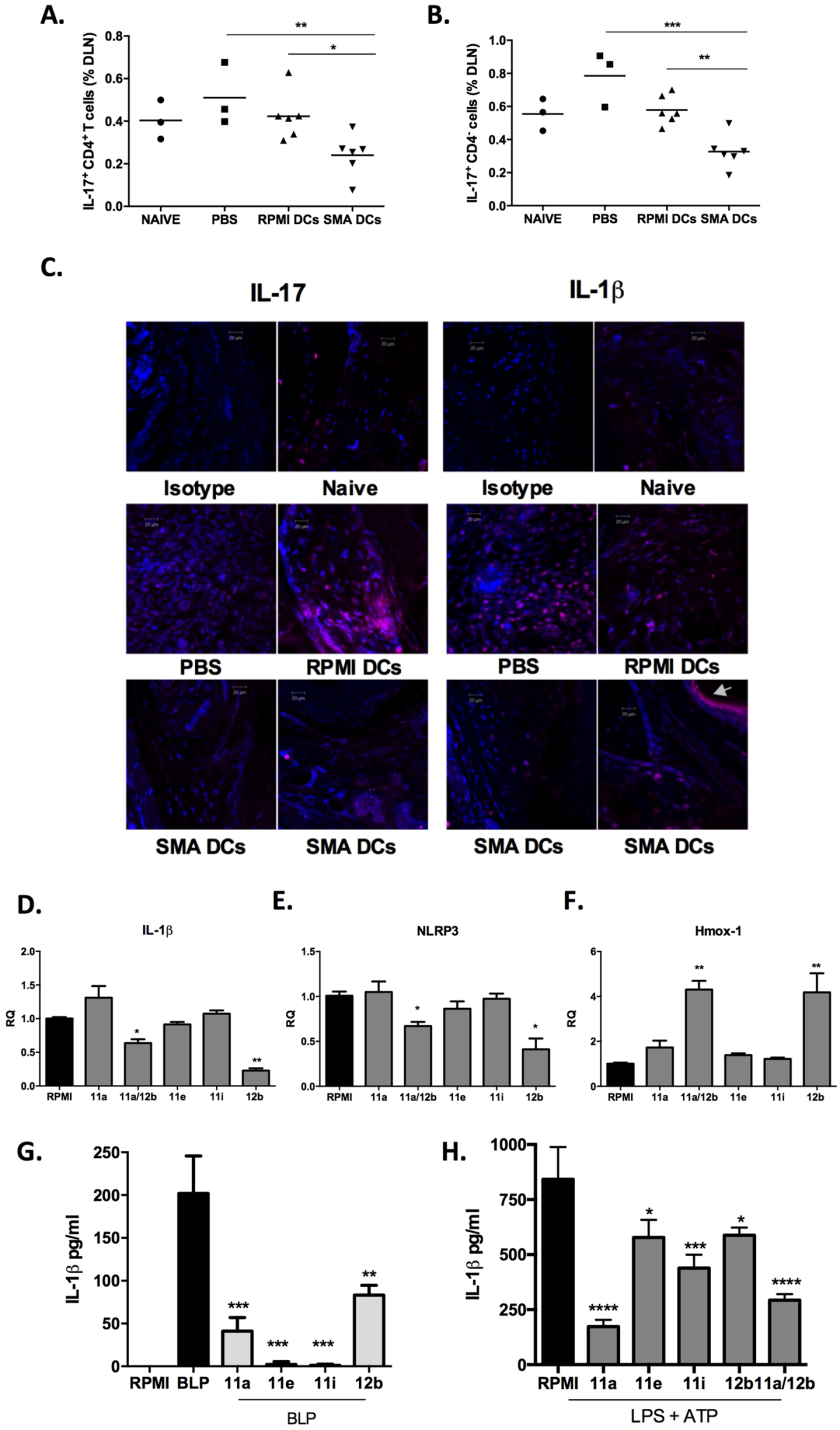
SMA-treated bmDCs inhibit IL-17 and IL-1β responses. The percentage of IL-17-expressing CD4^+^ cells (**A**) and IL-17-expressing CD4^−^ cells (**B**) in the DLNs from paws from naïve or CIA-mice treated with PBS, RPMI-DCs or SMA-DCs was determined by flow cytometry. Results from individual mice in each group (Naïve n = 3, PBS n = 3, RPMI-DCs n = 6 and SMA-DCs n = 6) are shown. Data are compared using one way ANOVA and Fishers LSD test where *p < 0.05 **p < 0.01. (**C**) Joint sections from individual mice representative of each treatment group were assessed for IL-17 and IL-1β expression by immunofluorescence (magnification 20x; scale bars 20 μm). The strong IL-1β positive staining in the SMA-DCs image (denoted by white arrow) reflects high production of IL-1β by keratinocytes in the skin and is an additional control for antibody specificity^40^. BmDCs were incubated with the indicated SMAs (5 μg/ml) for 4 hours, the RNA was extracted and the expression levels of IL-1β (**D**), NLRP3 (**E**) and HMOX-1 (**F**) measured by qRT-PCR. Cytokine expression was normalised to GAPDH and then expressed as a fold change with respect to the relevant RPMI control. The data shown are collated from 3 independent experiments and presented as means of mean values (triplicate assays in each experiment) ± SEM, n = 3. (**G**) BmDCs were incubated with the indicated SMAs (5 μg/ml) for 18 hours before stimulation with BLP for 24 hours and the levels of IL-1β measured by ELISA. Data are expressed as means (of triplicate determinations) ± SD. (**H**) BmDCs were incubated with the indicated SMAs (5 μg/ml) for 18 hours before being primed with LPS (100 ng/ml) for 5 hours then stimulated with 1 mM ATP for 30 minutes and the levels of IL-1β measured by ELISA. Data are expressed as means (of triplicate determinations) ± SD. Data in D-H were analysed using students t-test (or Wilcoxin signed rank test for normalised qRT-PCR data) where *p < 0.05, **p < 0.01, ***p < 0.001, ****p < 0.0001; data in G & H are from single experiments that are representative of at least two independent experiments.

As stated above, ES-62 mediates protection in CIA through the down-regulation of pro-inflammatory cytokines, in particular IL-17, and by targeting a complex network of cells, specifically through modulation of DCs^9, 12^ and thus the levels of IL-17-producing cells in the dLNs were analysed by flow cytometry. SMA DC-treated mice exhibited a significantly reduced proportion of IL-17^+^ CD4^+^ T cells in the dLNs compared to RPMI DC- and PBS-exposed mice (Fig. 5A). In addition, mice treated with SMA-DCs had a significantly reduced proportion of IL-17^+^ CD4^−^ cells that potentially represent γδ T cells in the lymphocyte gate, compared to mice treated with RPMI-DCs or PBS (Fig. 5B). Furthermore, SMA-DC treated mice had reduced levels of IL-17 staining in their joints compared to RPMI-DC and PBS-treated mice (Fig. 5C). There were no differences amongst the groups in the proportion of CD4^+^ T cells in the DLNs expressing IFNγ but a significant increase in the percentage of non-CD4^+^ cells expressing IFNγ was observed in PBS mice, and this was significantly decreased in mice inoculated with RPMI-DCs or SMA-DCs (Data not shown).

SMA 12b has been demonstrated to mediate its protective effect in CIA via suppression of IL-1β, rather than IL-17, responses^11^. Thus, it was investigated if protection afforded by SMA-DC treatment was also mediated through modulation of IL-β. Although no IL-1β could be detected in serum obtained from mice with CIA (results not shown), staining of IL-1β in the joint revealed that treatment with SMA-DCs greatly reduced expression of this cytokine compared to levels pertaining in mice treated with RPMI-DC or PBS (Fig. 5C). Supporting this finding, and consistent with our previous results in macrophages^11^, incubation with SMA 12b (or 11a/12b in combination) for 4 hours was found to inhibit the steady-state mRNA expression of IL-1β in DCs, an effect not noted with the other SMAs (Fig. 5D). Regarding mechanism, we have also previously shown in macrophages that SMA 12b acts to suppress IL-1β responses by reducing NLRP3 inflammasome expression in an NRF2-dependent manner^11^ and consistent with this, 12b and 11a/12b combination, but not 11a, 11e or 11i, similarly suppress NLRP3 (Fig. 5E) whilst inducing HMOX (a cyto-protective gene target of NRF2) gene expression in DCs over 4 hours (Fig. 5F). We also measured the release of IL-1β protein resulting from stimulation over 24 hours of DCs with BLP, a ligand of TLR2 signalling which has been proposed to play a key pathogenic role in the arthritic joint^18^ and found it to be reduced (Fig. 5G). Interestingly however, this effect was also noted with the other 3 SMAs, with 11e and 11i being particularly effective (Fig. 5G), perhaps suggesting that these SMAs may also target NLRP3 activation in DCs. In addition, it has recently been shown that PC can inhibit release of IL-1β from human and rat monocytes in response to LPS and ATP^26^, and similarly we found that all four SMAs could significantly reduce release of the cytokine from mouse bmDCs in response to this combination of stimuli (Fig. 5H). Here 11a was noted as being particularly potent, being as effective as 11a and 12b in combination. Recently it has emerged that the inflammasome is important in the development of IL-17 responses^27^, and therefore the SMAs may be mediating protection against CIA by targeting differing aspects of this interrelated response. Nevertheless, although not always reaching statistical significance, there is a trend for the SMAs other than 12b to exhibit similar effects on gene expression to the latter SMA when this is measured over 18 hours (results not shown).

## Discussion

Modulation of DC responses is a key mechanism by which ES-62 mediates many of its anti-inflammatory effects^6, 12, 20^ and here we demonstrate that four synthetic ES-62 SMAs - 11a, 11e, 11i and 12b, are similarly capable of targeting these cells. Studies *in vitro* revealed that like ES-62, the SMAs render bmDCs refractory to full activation by TLR PAMPs, as evidenced by decreased expression of co-stimulatory molecules and reduced release of pro-inflammatory cytokines. One difference we noted however, is that in contrast to ES-62, the SMAs have no obvious inhibitory effect on IL-12p40 protein production (in spite of 11e and 11i reducing mRNA production), but significantly suppress generation of IL-12(p70). We wished to gain some understanding of the molecular mechanisms underlying these effects and have previously found ES-62 to differentially modulate activation of MAPKs to mediate its anti-inflammatory effects on cytokine production. For example, whilst the nematode product induces ERK MAPK activation to negatively regulate IL-12p40 production, it suppresses the p38 activation required for generation of IL-12p35, IL-6 and TNF-α in macrophages. SMA 12b likewise significantly inhibited the LPS-induced activation of p38 in bmDCs consistent with its ability to suppress IL-12p35, IL-6 and TNF-α mRNA in these cells. However, it inhibited rather than activated LPS-induced ERK production and this may explain its failure to reduce IL-12p40 production. Moreover, ERK activation has been linked to T_H_2 responses and this signal transducer has been shown to be preferentially activated (phosphorylated) by helminth products, such as LNFPIII, a glycan found in soluble schistosome egg antigen (SEA), to help drive the T_H_2 response conducive to helminth survival^28^. Thus, as 12b suppressed LPS-induced ERK activation it is possible that it could target T_H_2 in addition to T_H_1 responses, although neither 11a nor 11i significantly inhibited such ERK activation despite 11a/11i-treated DCs tending to suppress Ag-specific T_H_2 responses in the *in vivo* adaptive transfer model. SMAs 11a and 11e also reduced ERK and p38 MAPK activation but this did not reach significance in all experiments. Instead, these SMAs, along with 12b, were found to significantly reduce the level of activation of NF-κB p65 in response to LPS. Inhibition of NF-κB has been found to retain DCs in an immature state^29, 30^, a mechanism used by other parasites including *F. hepatica* and *Brugia malayi* to modulate APC-priming of immune responses^31–33^. SMA 11i had no consistent effect on NF-κB p65 activation and, similar to 11a and 11e, reduced ERK and p38 MAPK but not significantly, suggesting that it may target other molecules to mediate its effects. Further understanding of how the SMAs mediate their inhibitory effects will require fully elucidating the nature of the signal transducers that they interact with, and also establishing whether like ES-62 they require a specific receptor for cell entry^34^.

*In vitro* exposure of DCs to SMAs also impacted on their activity *in vivo*. For example, pre-exposure of bmDCs to SMAs 11a or 11i before stimulation with LPS *in vitro* significantly inhibits the number of DLN Ag-specific IL-17^+^CD4^+^ and IFN-γ^+^CD4^+^ T cells generated compared to LPS-DCs following transfer of the DCs *in vivo*. This is likely a result of the reduced levels of IL-12, TNF-α and IL-6, key cytokines required for the differentiation of T_H_1/T_H_17 cells^35^, produced by these DCs compared to LPS-DCs. Suppression of T_H_17 responses by helminth-modulated DCs has, as far as we are aware, previously been reported solely by Dowling *et al*. who demonstrated that two molecules from *F. hepatica* ES only partially activate DCs, with consequent attenuation of OVA peptide-specific T_H_17 responses *in vivo* ^36^. Interestingly neither of these molecules induced DCs to drive T_H_2 responses *in vivo*, results, which are similar to our data with SMA-modulated DCs. Indeed, none of the SMAs appeared to promote the production of IL-4 either *in vitro* or *in vivo* suggesting that while they can suppress T_H_1/T_H_17 responses, like ES-62, they may not actually drive the ‘opposing’ T_H_2 phenotype (if anything, the opposite).

The use of *in vitro* helminth-modulated DCs to treat or ameliorate disease has been demonstrated in several murine models of inflammatory disorders suggesting that DCs can play a central role in regulating the pathogenesis of autoimmune diseases such as arthritis, MS and colitis^13–15^. Here we demonstrate that transfer of bmDCs treated *in vitro* with 11a plus 12b protects against the development of inflammatory CIA in recipient mice. Of the four sulfones found to modulate DC responses, SMAs 11a and 12b were chosen for this model as their protective effect in CIA has already been established: although both SMAs act by mimicking ES-62 in downregulating MyD88 expression to subvert IL-1R/TLR responses, 11a appears primarily to mediate protection through suppression of downstream IL-17 responses while 12b acts predominantly to limit IL-1β production by targeting the inflammasome via counter-regulatory NRF2 signalling^9, 11^. Thus, it was hypothesised that employing them in combination to target two pathogenic axes, could maximise any potential protective effects in this model. Protection was indeed observed and was associated with significantly lower levels of IL-17^+^CD4^+^ cells and IL-17^+^CD4^−^ cells in the dLNs compared to PBS- and RPMI DC-treated mice, as well as reduced IL-17 and IL-1β expression in the joint. IL-17 was also found to be decreased in the joints of mice treated with DCs matured with *F. hepatica* total extract and CpG (FTegDCs) and dLN cells from these mice showed reduced levels of IFN-γ and IL-17 when stimulated *ex vivo* with type II collagen (CII)^13^. This indicates that suppression of IL-17 responses may be a common mechanism by which helminths can ameliorate experimental models of arthritis. This could reflect that the induction of IL-10-producing T regulatory (Treg) cells appears to be a key mechanism utilised by several helminths to prolong their survival within the host^37^ and certainly, there are reports that helminth-matured DCs can modulate inflammatory disease via an increase in IL-10 or generation of Treg cells. For example, adoptive transfer of DCs matured with *Hymenolepis diminuta* antigen confers protection against the development of experimental colitis and this is dependent on the production of IL-10 by cells of the adaptive immune system^15^. Also, the protection afforded by FTegDCs was associated with an increase in FOXP3^+^CD25^+^CD4^+^ Treg cells, which were able to confer protection when transferred into recipient mice^13^. In contrast to these models of immunomodulation by helminth infection/helminth Ag, we found no differences in the proportion of IL-10^+^FOXP3^+^CD4^+^, IL-10^+^CD4^+^ or IL-10^+^CD4^−^ cells in the dLNs of any of the mice indicating that this is not a mechanism employed by SMA-treated DCs to down-regulate CIA. This is consistent with previous findings with ES-62, which has consistently been found not to promote Treg responses but rather to act on effector cells to suppress inflammatory responses^12, 20, 24^. However, ES-62 has also previously been shown to induce protective IL-10-producing Breg cells^25^ and, although we did not examine effects on the various reported Breg populations directly in this present study, we did not observe an increase in IL-10 production and expression in draining lymph node CD4^−^ cells: if IL-10^+^ regulatory B cells (B10 cells) had been affected, we might have expected to observe some increases in this population. Hence, we can only assume therefore that such cells are not generated via the DC-T cell interactions that would be promoted by adoptive transfer of SMA-pulsed DCs. ES-62 SMA-mediated protection was also not associated with a difference in antibody responses between SMA DC-treated mice and PBS or RPMI DC-treated mice despite ES-62 being previously shown to skew the humoral immune response from an IgG2a-dominant to IgG1-dominant anti-CII response^23^. However, PC-BSA, which has been shown to mimic ES-62-mediated protection in CIA, did not have any effect on antibody responses^38^.

Collectively, our data highlight the importance of DCs in regulating pathology in CIA and also provide insight into the mechanism of SMA-mediated protection as the modes of action (targeting IL-17 and IL-1β production) of the SMA-DCs closely mimic the mechanisms by which direct administration of these SMAs protects mice from CIA. In addition, these data demonstrate that, despite ES-62 and the SMAs having multiple potential targets within the immune system^9, 11, 12^, many of their effects can simply be recapitulated by transfer of DCs exposed to these products. Thus, in addition to adding to our knowledge of the mechanism of SMA-mediated protection, these data pinpoint DCs as a potential therapeutic target in RA. This is potentially highly useful information, as the drugs available against RA remain inadequate: for example, although cytokine blockers such as infliximab (targeting TNF-α) have had some success, the number of patients achieving remission remains low^8^. Thus, new therapeutics are required, and the SMAs, due to their anti-inflammatory properties, allied to the ease and low cost of their production, are attractive drug candidates. Once again, it must be emphasised that it is important to understand how they function, the cells they target, and ultimately the optimum structure of compound to employ. Compound optimisation will necessarily take into account pharmacokinetics, toxicity, and metabolism. As things stand, all four SMAs selected from the *in vitro* cytokine screen are sulfones (Structures in Fig. 1), indicating that replacing phosphorus with sulphur (which results in intrinsically non-hydrolysable isosteres of phosphates) does not prevent the generation of compounds that mimic the anti-inflammatory effects of ES-62’s PC group. The four compounds each contain a two-carbon methylene chain between the sulfone group and choline derivative but vary slightly in their aromatic substituents and secondary amide/choline derivative and this may help explain the subtle differences in terms of their immunomodulatory effects. Specifically, 11a and 12b contain bromine and a methyl group respectively at C4 on their aromatic ring while 11e and 11i contain fluorine at C3 and C4 respectively. In addition, 11a, 11e and 11i are all tertiary dimethyl amines while 12b is a quaternary ammonium salt, making it permanently positively charged, which would be expected to have a significant impact on 12b’s ability to interact with receptors and gain access to the cell and intracellular compartments^11^. Clearly as alluded to earlier, further consideration/analysis is required in this area but ultimately it may be possible to explain such observations as to why 12b targets ERK and p38 MAPKs whilst the other SMAs do not.

In conclusion, we have demonstrated the anti-inflammatory effects of SMAs of the helminth product ES-62 on DCs *in vitro* and *in vivo* and show that treatment with SMA-treated DCs is sufficient to prevent the development of CIA in mice. These data therefore highlight the therapeutic potential of DCs as a key target cell for SMA-mediated protection against disease.

## Methods

### Animals

Unless indicated otherwise, all mice were specified pathogen-free and maintained under standard *ad libitum* conditions at the Universities of Strathclyde and Glasgow SPF Biological Services Facilities. All experimental procedures were approved by, and performed in accordance with, the Animal Welfare and Ethical Review Body at the University of Glasgow, the Ethical Review Board of the University of Strathclyde and UK Home Office Regulations and Licenses PPL 60/4300 and PIL 60/4554. Male 6–8 week-old BALB/c or C57BL/6 mice were used to generate bone-marrow derived dendritic cells (bmDCs) and were bred at the University of Strathclyde for all experiments except the adoptive transfer experiment where the mice were purchased from Charles River Laboratories (Tranent, Scotland). For the *in vitro* co-culture experiments and *in vivo* adoptive transfer experiments, mice homozygous for the transgenic TCR which is specific for a chicken ovalbumin peptide (OVA_323–339_) in the context of I-A^d^ (D0.11.10 on a BALB/c background) were used as T cell donors^22^. Collagen (CII)-induced arthritis (CIA) was induced in male DBA/1 mice (10 weeks; Harlan Olac; Bicester, UK) and these mice were also used to generate bmDCs for use in the CIA study. DBA/1 animals were maintained in the Biological Services Unit of the University of Glasgow in accordance to the UK Home Office Licences PPL 60/4314, PIL IC9B4F104 and PIL I675F0C46 and the Ethics Review Board of the University of Glasgow. Arthritis was induced by intradermal immunization with CII emulsified with complete Freud’s adjuvant (MD Biosciences) on day 0 (injection volume 100 μl) and with CII in PBS intraperitoneally on day 21 (injection volume 200 μl) and scored for development of arthritis as previously described^23^. Mice were inoculated with bmDCs that had either been treated with SMAs for 18 hours or maintained in complete RPMI 1640 (containing 2 mM glutamine, 50 U/ml penicillin, 50 µg/ml streptomycin and 10% FCS; cRPMI) alone, on days −2, 0 and 21. Animals were culled on day 35 and the blood, paws and popliteal draining lymph nodes (dLNs) harvested for analysis.

### *In vitro* cell culture

BmDCs were prepared essentially as described previously^39^. Briefly, bone marrow was obtained from the femurs and tibias of BALB/c mice and cells grown in cRPMI 1640 supplemented with 10 ng/ml GM-CSF (PeproTech, London, UK) at 37 °C in 5% CO_2_ for 8 days with fresh medium supplemented with GM-CSF added on days 3 and 6. On day 8 loosely adherent cells were harvested and found to be 75–85% CD11c^+^MHC II^+^, as determined by flow cytometry. Such cells were employed as bmDCs and were treated with SMAs (5 μg/ml) for 18 hours before stimulation with PAMPs: LPS (100 ng/ml or 1 μg/ml; *Escherichia coli* 055:B5, Sigma-Aldrich); BLP (100 ng/ml; Pam_3_CSK_4_, InvivoGen, Toulouse, France) and CpG (0.1 μM; Sources Biosciences, Nottingham, UK) for 24 hours and the supernatants recovered for cytokine analysis. Additionally, BmDCs were pre-treated with SMAs (5 μg/ml) for 18 hours, primed with LPS (100 ng/ml) for 5 hours and stimulated with ATP (Sigma-Aldrich) for 30 minutes and the supernatants recovered for IL-1β analysis. For bmDC-T cell co-cultures, bmDCs were incubated with SMAs for 18 hours, matured with LPS for 24 hours and then pulsed with OVA_323–339_ peptide (0–300 nM) before incubation with naïve T cells derived from OVA-specific DO.11.10 BALB/c mice for 3 days. Naïve T cells were isolated using Miltenyi magnetic bead technology, as CD62L^+^CD4^+^ T cells.

### ELISA

Interleukin-6 (IL)-6, IL-12p40, TNF-α (all BD Pharmingen, Oxford, UK), IL-12p70, IFN-γ, IL-4 (all eBioscience, Hatfield, UK) and IL-17 and IL-1β (R&D Systems, Abingdon, UK) secretion was measured using enzyme-linked immunosorbent assays (ELISAs) according to the manufacturers' instructions. The cytokines were detected using biotinylated monoclonal antibodies, streptavidin horseradish peroxidase (SAv-HRP) and TMB substrate.

### Flow cytometry

Expression of costimulatory molecules on DCs was quantified by flow cytometry using anti-CD11c-Pe/Cy7, anti-CD86-PerCP (both BioLegend), and biotinylated anti-CD40 (eBioscience; detected by streptavidin-APC/Cy7, BD Pharmingen) antibodies, with the gating strategy shown in Supplementary Figure 1. Popliteal DLN cells (10^6^/ml) from *in vivo* models were incubated ± 50 ng/ml PMA plus 500 ng/ml ionomycin for 1 h before addition of 10 µg/ml Brefeldin A (Sigma-Aldrich, UK) for a further 5 h at 37 °C with 5% CO_2_. Phenotypic markers were labelled using anti-CD4-FITC or anti-CD4-PerCP (both BD Bioscience) and anti-KJ1.26-APC (eBioscience) before the cells were fixed and permeabilized employing the FOXP3 Fix/Perm buffer set according to eBioscience protocols^40^. Cells were then labelled using anti-IL-17A-PerCP or APC/Cy7, anti-IFN-γ-APC or PeCy7, anti-IL-4-PE, anti-IL-10-PE and anti-FOXP3-APC (all eBioscience) antibodies for 30 min prior to flow cytometry. Data were acquired using a FACSCanto immunocytometry system (BD Pharmingen) with samples gated according to appropriate isotype and fluorescence minus one controls and analysed using FlowJo software (Tree Star Inc, OR, USA, version 7.6.1). The gating strategy for the adoptive transfer model is shown in Supplementary Figure 2 whilst those for analysis of DLN cells from CIA mice are shown in Supplementary Figures 3 and 4.

### qRT-PCR

BmDCs were treated with SMAs (5 μg/ml) for 18 hours and then matured with LPS (100 ng/ml) for 4 hours before extraction of RNA using the RNeasy Plus Mini kit from Qiagen (Hilden, Germany) according to the manufacturer’s instructions. RNA was transcribed using the High Capacity cDNA Reverse Transcription kit (Applied Biosystems, Life Technology) and duplicate PCR amplifications were performed using the StepOne Plus™ real-time PCR system according to the manufacturer’s instructions (Applied Biosystems). Applied Biosystems assay kits for IL-6 (Mm00446190_m1), TNF-α (Mm00443259_g1), IL-12p35 (IL-12a, Mm00434165_m1), IL-12p40 (IL-12b, Mm00434174_m1), IL-1β (Mm00434228_m1), NLRP3 (Mm04210224_m1) and HMOX-1 (Mm00516005_m1) were used. Data were analysed by RQ Manager software (Applied Biosystems) and normalized to the reference reporter glyceraldehyde 3-phosphate dehydrogenase (GAPDH, Mm99999915_g1).

### Signalling Assays

BmDCs were treated with SMAs (5 μg/ml) for 18 hours, then matured with LPS (100 ng/ml) for 30 minutes. Nuclear cell extracts were obtained using the Nuclear Extract kit and the levels of NF-κBp65 in the nucleus determined using a TransAM NF-κBp65 Transcription Factor Assay kit according to the manufacturer’s instructions (Actif Motif, LaHulpe, Belgium). Likewise, for Fast Activated Cell-Based ELISA (FACE) assays, bmDCs were treated with SMAs (5 μg/ml) for 18 hours, then stimulated with LPS (100 ng/ml) for 10 minutes on 96-well plates that were pre-coated with Poly-l-Lysine (Sigma-Aldrich). Cells were fixed in 4% (w/v) paraformaldehyde (Santa Cruz Biotechnology, Texas, USA) and the expression of total and phosphorylated forms of ERK and p38 MAPKs determined as described previously^7^.

### Analysis of joint pathology and cytokine expression in the joints

Representative paws from each group in the CIA model were used to examine joint pathology and cytokine expression. Decalcified joint tissue section preparation, Trichrome staining and cytokine detection by immunofluorescence were undertaken as previously described^11, 12, 40^. To detect cytokine expression, the joint sections were de-waxed in Xylene, and re-hydrated by incubation with decreasing concentrations of ethanol before incubation in citrate buffer (10 mM citric acid, 0.1% Tween, pH 6.0) for antigen retrieval. Sections were then blocked against endogenous biotin (Molecular Probes, Oregon, USA) and incubated with goat anti-mouse IL-17 antibody (Abcam, Cambridge, UK) or rabbit anti-mouse IL-1β antibody (Abcam) for 12 h at 4 °C. Signal was detected using biotinylated secondary antibodies (anti-goat IgG for IL-17 and anti-rabbit IgG for IL-1β) and Streptavidin-Alexa Fluor647. Sections were then dehydrated by incubation in increasing concentrations of ethanol, mounted and counterstained using VECTORSHIELD mounting medium containing DAPI (Vector Laboroatories, California, USA). Immunofluorescence images were obtained using an LSM 510 META confocal laser coupled to an Axiovert 200 microscope (Zeiss) and analysed using Zeiss LSM Image Browser software (Oberkocken, Germany).

### Statistical Analysis

Parametric data were analysed by Student t test, one-way ANOVA with Bonferroni post-test or Fishers LSD test. Non-parametric data were analysed using Mann-Whitney U or Kruskal-Wallis with Dunn’s post-test. In all cases *p < 0.05, **p < 0.01 and ***p < 0.001.

## Electronic supplementary material

Supplementary Information
