# Supplementary material for: Dendritic cells provide a therapeutic target for synthetic small molecule analogues of the parasitic worm product, ES-62
Source: Sci Rep. 2017 May 10;7:1704. doi: 10.1038/s41598-017-01651-1 (PMC5431997; doi:10.1038/s41598-017-01651-1)
Supplement: Supplementary file 1 — Supplementary Information [file 41598_2017_1651_MOESM1_ESM.pdf]

Dendritic cells provide a therapeutic target for synthetic small molecule analogues of the parasitic worm product, ES-62

Felicity E. Lumb[1], James Doonan[1], Kara S. Bell[1], Miguel A. Pineda[2], Marlene Corbet[2], Colin J. Suckling[3], Margaret M. Harnett[2*] and William Harnett[1*]

[1]Strathclyde Institute of Pharmacy and Biomedical Sciences, University of Strathclyde, Glasgow, UK , [2]Institute of Infection, Immunity and Inflammation, University of Glasgow, Glasgow, UK and [3]Department of Pure & Applied Chemistry, University of Strathclyde, Glasgow UK

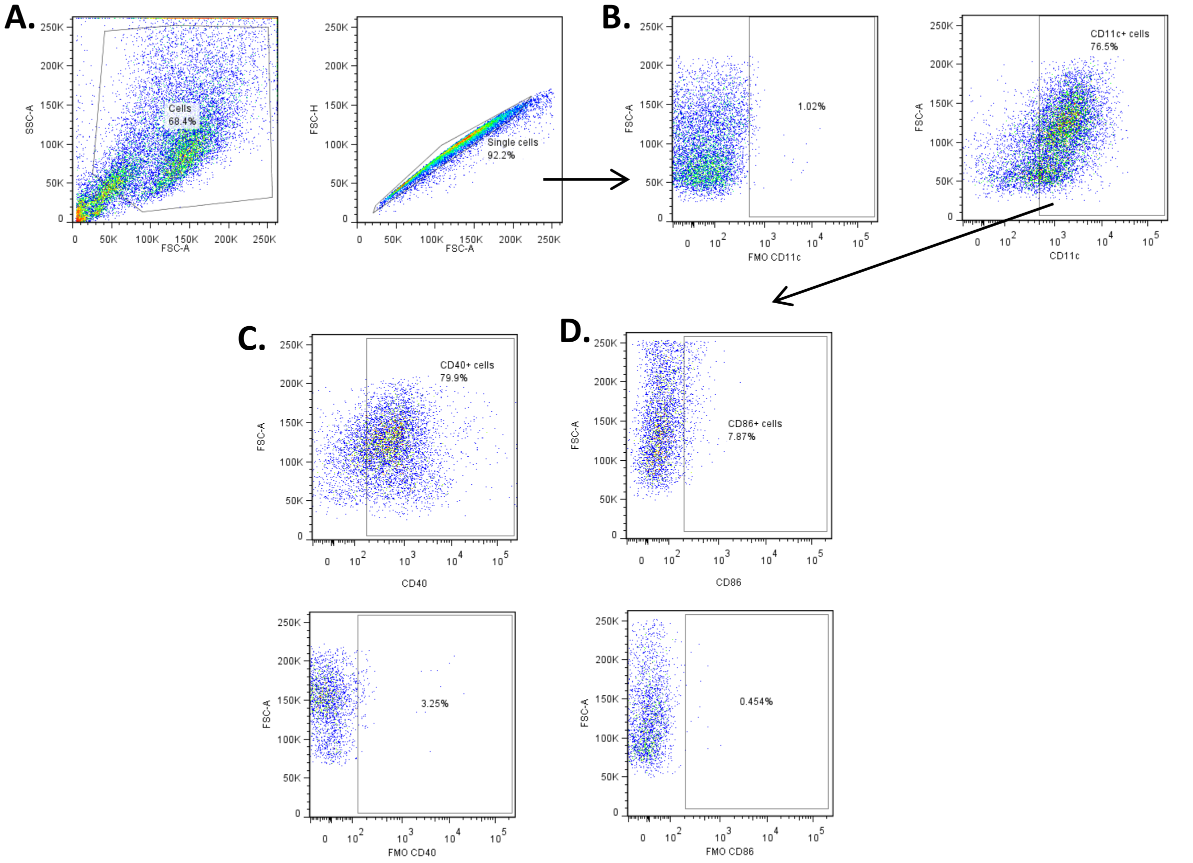

Figure 1: Gating strategy for investigation of co-stimulatory molecule expression by $CD11c^+$ cells. Cells were initially gated based on size (forward scatter; FSC) and granularity (side scatter; SSC) and doublets were excluded by comparing FSC-Height and FSC-Area (A). Fluorescent minus one (FMO) control analysis was used to determine the expression of $CD11c^+$ (Pe/Cy7) cells (B). The expression of CD40 (APC/Cy7) (C) and CD86 (PerCP) (D) on these $CD11c^+$ cells was then determined relative to their FMO controls.

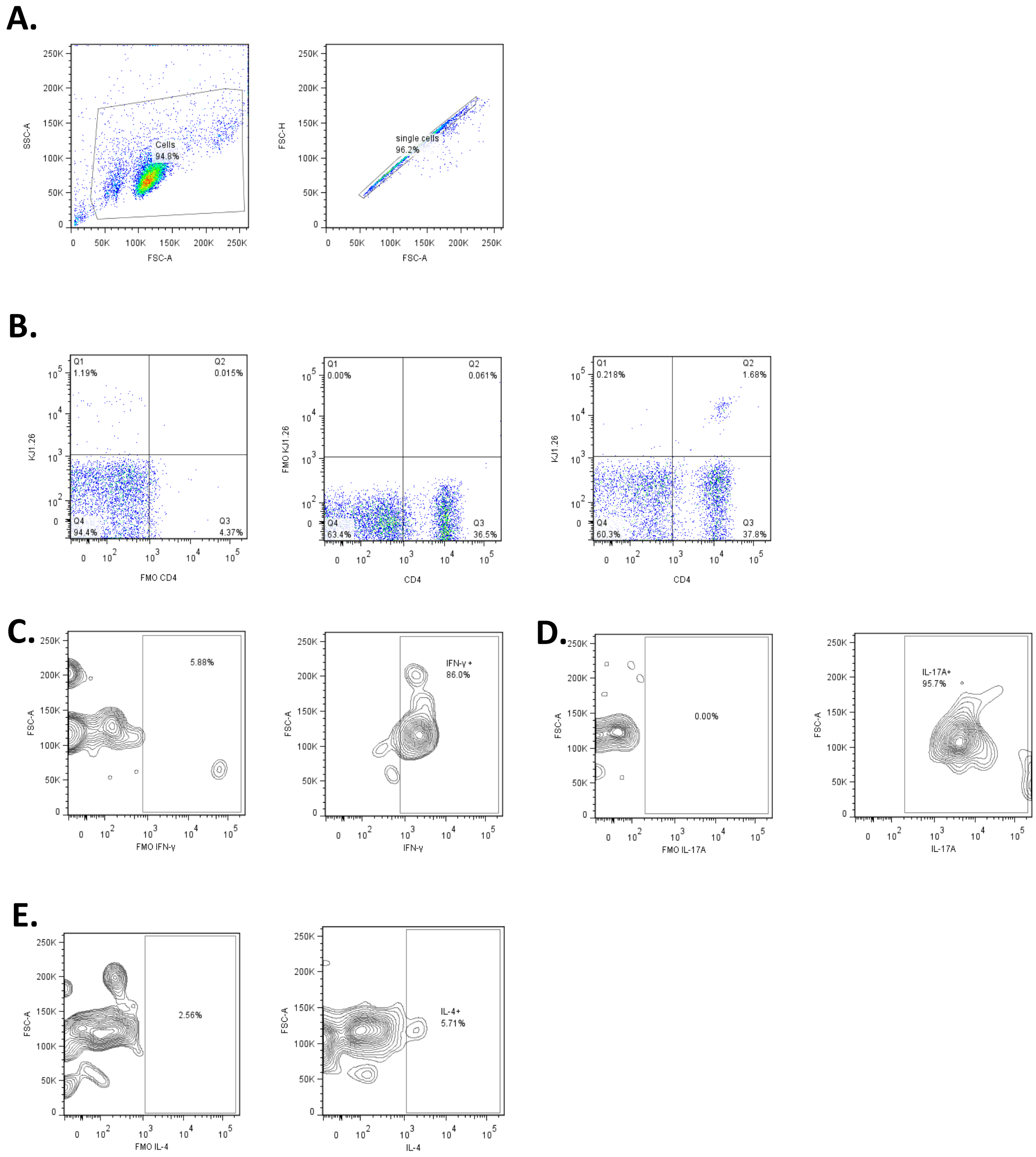

Figure 2: Gating strategy for the identification of CD4$^+$KJ1.26$^+$ T cells. Cells were stimulated with PMA and Ionomycin for an hour before the addition of Brefeldin A for a further 4 hours. Cells were initially gated based on size (forward scatter; FSC) and granularity (side scatter; SSC) and doublets were excluded by comparing FSC-Height and FSC-Area (A). Fluorescent minus one (FMO) controls were used to determine the expression of CD4$^+$ (PerCP) and KJ1.26$^+$ (APC) cells (B). The expression of IFN-γ (Pe/Cy7) (C), IL-17 (APC/Cy7) (D) and IL-4 (PE) (E) on these CD4$^+$KJ1.26$^+$ T cells was then determined, also using FMO controls.

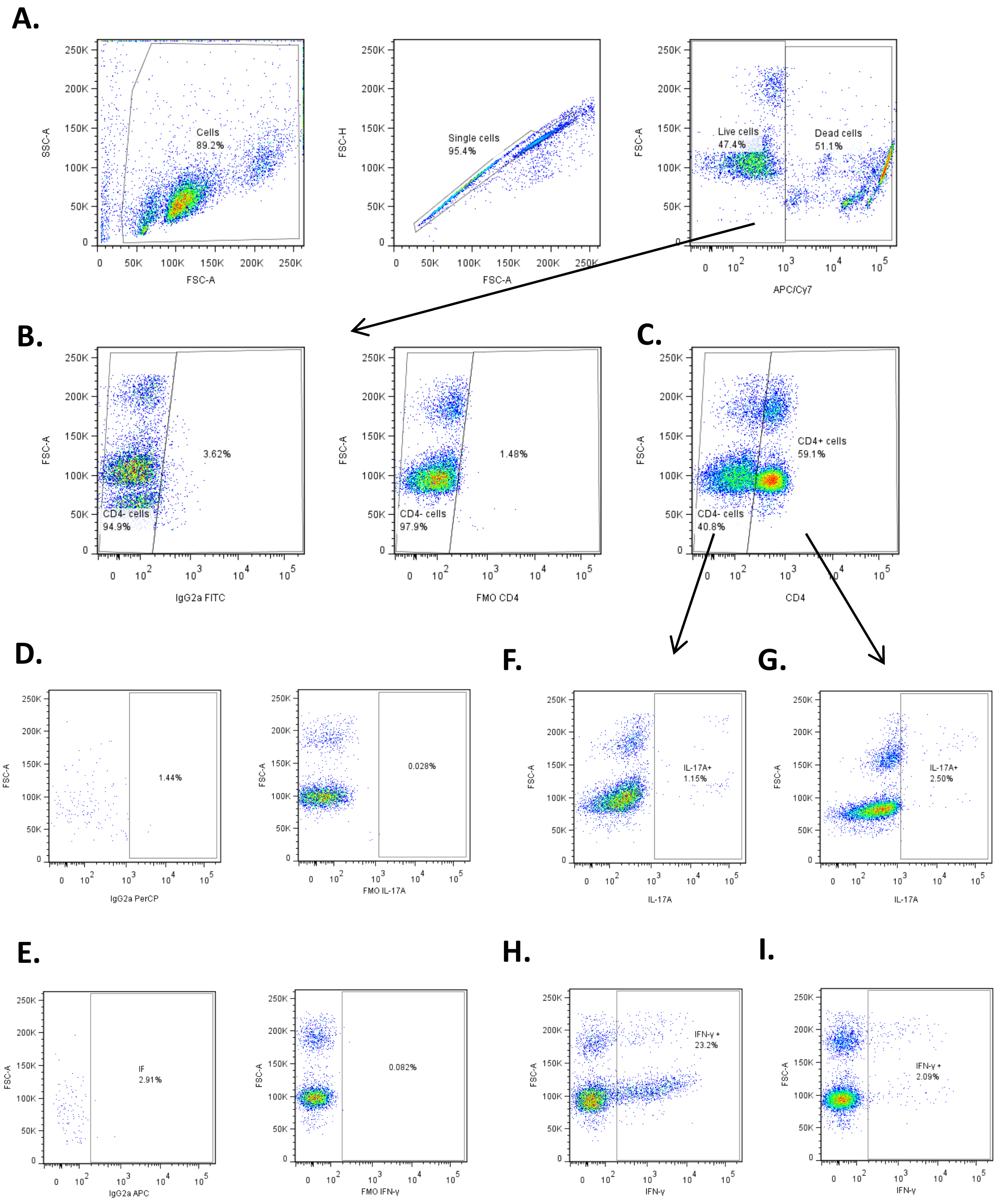
A.
B.
C.
D.
E.
F.
G.
H.
I.
SSC-A
FSC-A
FSC-H
Cells
89.2%
Single cells
95.4%
Live cells
47.4%
Dead cells
51.1%
APC/Cy7
CD4- cells
94.9%
3.62%
IgG2a FITC
CD4- cells
97.9%
1.48%
FMO CD4
CD4+ cells
59.1%
CD4- cells
40.8%
CD4
1.44%
IgG2a PerCP
0.028%
FMO IL-17A
IL-17A+
1.15%
IL-17A+
2.50%
IL-17A
IF
2.91%
IgG2a APC
0.082%
FMO IFN-γ
IFN-γ +
23.2%
IFN-γ +
2.09%
IFN-γ

Figure 3: Gating strategy for the identification of CD4$^+$ and CD4$^-$ cells in the dLNs of CIA mice. Cells were stimulated with PMA and Ionomycin for an hour before the addition of Brefeldin A for a further 4 hours. Cells were then stained with the Fixable Viability Dye eFluor® (APC/Cy7) to exclude dead cells and for expression of CD4 (FITC) prior to permeabilisation and staining for IL-17A (PerCP) and IFN-γ (APC). Initially cells were gated based on size (FSC) and granularity (SSC), doublets were excluded by comparing FSC height and FSC area and the Fixable Viability Dye eFluor® (APC/Cy7) used to exclude dead cells (A). Relevant isotype and fluorescent minus one (FMO) controls (B) were used to determine the expression of CD4 on lymphocytes (C). Likewise, relevant isotype and FMO controls (D and E) were used to determine the expression of IL-17A by CD4$^+$ (F) and CD4$^-$ (G) T cells, and the expression of IFN-γ by CD4$^+$ (H) and CD4$^-$ (I) T cells.

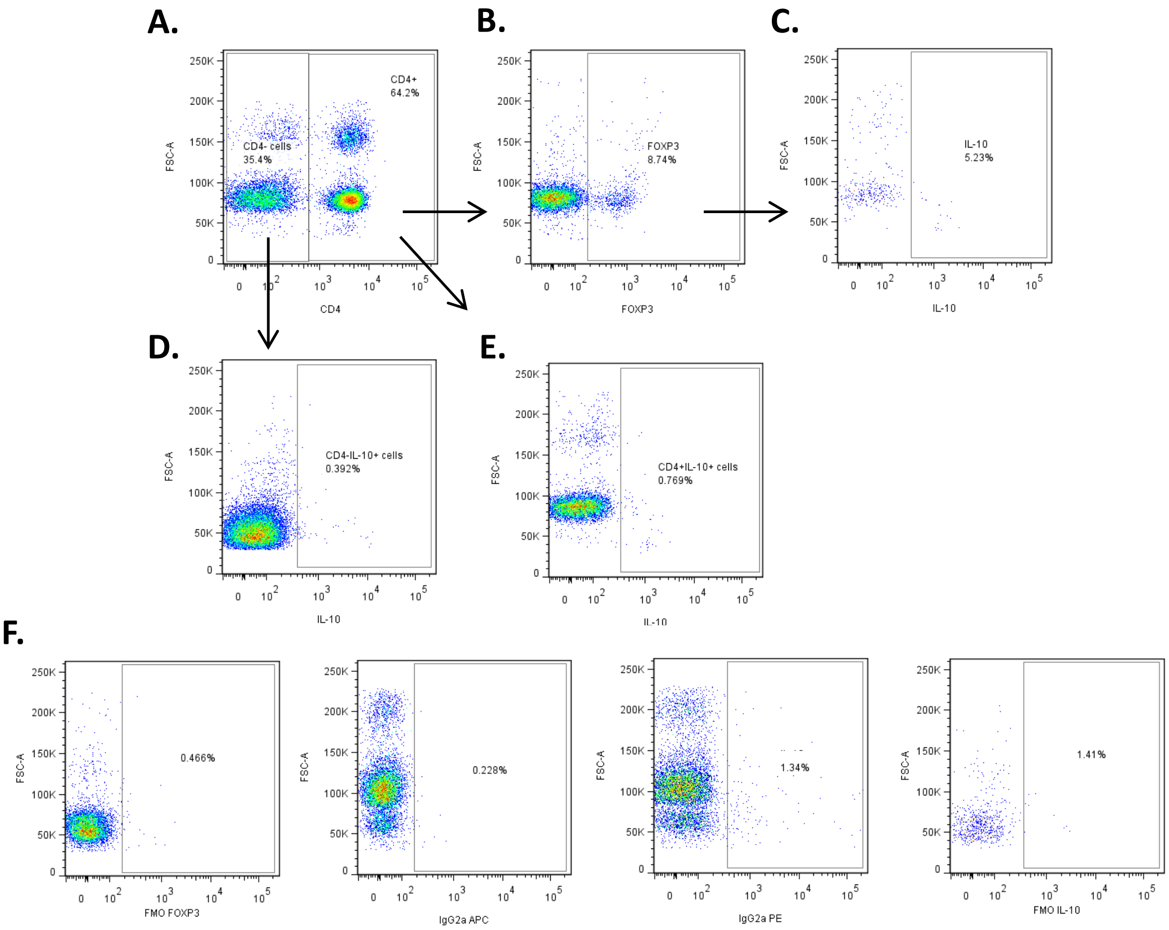

Figure 4: Gating strategy for the identification of regulatory cells in the dLNs of CIA mice. Cells were stimulated with PMA and Ionomycin for an hour before the addition of Brefeldin A for a further 4 hours. Cells were then stained with the Fixable Viability Dye eFluor® (APC/Cy7) to exclude dead cells and for expression of CD4 (PerCP), prior to permeabilisation and staining for expression of FOXP3 (APC) and IL-10 (PE). Cells were initially gated as described in Supplementary Figure 3A and relevant isotype and FMO controls were used to determine the expression of FOXP3 on CD4$^+$ cells (B) and IL-10 by these CD4$^+$FOXP3$^+$ cells (C). These controls were also used to determine the expression of CD4$^-$IL-10$^+$ cells (D) and CD4$^+$IL-10$^+$ cells (Tr1 cells) (E). The Isotype and FMO controls for FOXP3 and IL-10 are shown in F.

SMA	IL-6			TNF-α			IL-12p40		
	1	2	3	1	2	3	1	2	3
11a	ND	32	68		76		187	163	
11e	ND	18	ND	38	35	50			
11f							41		
11g		67		36	61		40		
11h	64		61	51	68		45		
11i	ND	ND	30	49	34	48	56		
11j		40	53						
11k		60	54	39	60		54		
11l									
11n				55					
12b	ND	24		31		95		139	
18a									
18b			78			63			
19a			x			x			x
19b						74			
19c									
19d									
19e				32		73			
19f									
19g									
19i									
19k									
19l									
19p									
19q									
19r					149	65			
19s									
19t									
19o			x			x			x
19u									
19v									
19w									
19z	57			73		62	68		
19aa	41			70		44	67		
21a									
21c									
21d			65						
21f			161						
21g			152						
21h									
21i									

SMA	IL-6			TNF-α			IL-12p40		
	1	2	3	1	2	3	1	2	3
21j					43				
21k		68							
21l									
21n		71							
21o			57						
21p									
21q									
23a									
23b									
23c	78		71						
23g			39			38			
24a	75								
24b			66						
24c	50								
24d				52					
24e	42		65						
25a									
25b									
25c									
25d						152			
62	52					56			
63		42			52				
64					68				
66					50				
67					63				
70					70				
72	50	47			44				
75	51				82				
80					77				
81					68				
82					84				
83					71				
84					76				
85									
88		64			78		57		
97		35			53				
98									
100									

Supplementary Table 1 Legend: The immunomodulatory effect of ES-62 SMAs on cytokine production by bmDCs exposed to LPS

The percentage of IL-6, TNF-α, and IL-12p40 produced as a result of pre-treatment with each of the selected SMAs compared to the LPS control for each of 3 experiments is shown. Only statistically significant values are shown, with blank boxes corresponding to non-significant changes. ND (not detected) is used in cases where SMA pre-treatment resulted in cytokine levels below the detection levels of the ELISA kit (≥ 15 pg/ml) and X signifies that no experiment was undertaken.
